# Clinical factors associated with intestinal strangulating obstruction and recurrence in adhesive small bowel obstruction: A retrospective study of 288 cases

**DOI:** 10.1097/MD.0000000000012011

**Published:** 2018-08-24

**Authors:** Jian-Feng Mu, Quan Wang, Shi-Dong Wang, Chuan Wang, Jia-Xing Song, Jing Jiang, Xue-Yuan Cao

**Affiliations:** a Department of Gastric and Colorectal and Anal Surgery; b Division of Clinical Research; c Clinical Laboratory, The First Hospital of Jilin University, Changchun, Jilin Province, China.

**Keywords:** adhesive small bowel obstruction, clinical parameter, follow-up, intestinal strangulation, recurrence

## Abstract

Postoperative adhesions are a common cause of adhesive small bowel obstruction (ASBO), and recognition of intestinal strangulation is important. The aim of this study is to analyze the clinical factors for strangulating obstruction and to identify the predictors for recurrence of ASBO.

A retrospective study was conducted using the database in our department. Patients with ASBO from January 2013 to April 2016 were included in the study and were subject to follow-up. The clinical factors associated with strangulating obstruction and recurrence after treatment were analyzed by using univariate and multivariate logistic regression model.

In total, 288 ASBO patients were included in the study. Of these, 37 (12.9%) patients had occurred strangulating obstructions, and 251 (87.1%) patients had simple obstructions. Four clinical parameters, including increasing heart rate (>100 bpm), increasing WBC count (>15 × 10^9^/L), CT findings of thickening or swelling of the mesentery, and CT showing seroperitoneum were detected as independent clinical factors for intestinal strangulation. Eighty-four (29.2%) patients experienced recurrence of obstruction during the median 24 months of follow-up. Recurrence rates were reduced in patients who underwent surgical treatment compared with those who received conservative management [21.3% (26/122) vs 34.9% (58/166) (*P* = .010)]. Nevertheless, the recurrence rates were not significantly increased in patients with strangulating obstructions compared with those with simple ASBO [34.3% (12/35) vs 27.7% (72/253) (*P* = .186)].

Four clinical parameters including tachycardia, leukocytosis, along with CT findings of thickening or swelling of the mesentery and CT showing seroperitoneum, associated with occurrence of intestinal strangulation in ASBO. ASBO patients who underwent surgical treatment had a reduced recurrence rate, but ASBO patients with strangulating obstructions had not increase the recurrence rates than those of patients with simple ASBO.

## 1 Introduction

Intestinal obstruction is one of the most common abdominal emergencies and has a complicated pathogenesis and variable prognosis; death can be the most serious result. Adhesive small bowel obstruction (ASBO) is generally caused by postoperative adhesions, and ASBOs are responsible for 60% to 75% of small bowel obstructions.^[1]^ The mortality of ASBOs range between 2% and 8%, and the proper management of ASBO is a clinical challenge for surgeons.^[2]^ The main cause of failure to cure ASBOs is due to delays in initial diagnosis and surgical treatment for strangulating obstructions. Controversy remains as to which patients with ASBO need immediate surgery; thus, how to distinguish a strangulating obstruction from a simple ASBO is very important. Moreover, many patients with ASBO have a recurrence after conservative management or surgery. Conservative treatment may leave adhesions that could cause the recurrence of obstruction. Operative management can lead to the formation of new adhesions, which in turn can contribute to recurrence.^[3,4]^ Therefore, the treatment methods (operative or conservative) may influence the risk of recurrence.^[5,6]^

In the present study, after long-term follow-up of patients with ASBO treated with operative and conservative management, we evaluated the clinical parameters associated with strangulating obstruction and the risk factors with recurrence rates for ASBO.

## 2 Materials and methods

### 2.1 Patient collection

Three hundred and ninety-eight consecutive cases of SBO were collected from January 2013 to April 2016 by using the database from the Department of Gastric and Colorectal and Anal Surgery, at the First Hospital of Jilin University. The medical records, including data for symptoms and signs, laboratory tests, and imaging examinations, were reviewed. Written informed consent was obtained from each patient, and the study protocol was approved by the Ethics Committee of the First Hospital of Jilin University.

### 2.2 Diagnosis and exclusion criteria

In this study, initial evaluation for ASBO patients was as follows: history of abdominal surgery; presenting symptoms of abdominal pain and distension, nausea, vomiting, or a lack of flatus and defecation; physical examination showing abdominal swelling with intestines with palpable peristaltic waves, tenderness, changing bowel sounds, or peritonitis. Based on the part of clinical findings, the inclusion criteria for ASBO should be met one of the following conditions: plain abdominal radiographs or abdominal ultrasound showing definite multiple air-fluid levels in the small bowel but no evidence of gas within the colon; CT or MRI imaging examination showing of small bowel obstruction; confirmation of ASBO by laparotomy or laparoscopy.

The exclusion criteria were as follows: intestinal obstruction secondary to colon cancer, small bowel carcinoma, gastrointestinal stromal tumors (GIST), or metastatic tumors; history of intra-abdominal thermotherapy or abdominal radiotherapy; obstruction caused by stool mass; abdominal tuberculosis; and early postoperative obstruction within 1 month. The diagnosis of intestinal strangulation was made during laparotomy assessment.

### 2.3 Clinical parameters and follow-up

Clinical parameters collecting included the following: general state: sex, age, history of surgery and abdominal trauma; symptoms and physical signs: fever, increasing heart rate, persistent abdominal pain, weakening or disappearing bowel sounds, and peritonitis; laboratory examination: peripheral blood white blood cell count and aspartate aminotransferase (AST) values; imaging examination: CT findings of decreasing intestinal wall density and thickening or swelling of the mesentery, and abdominal cavity effusion (seroperitoneum).

Indications for surgical intervention were based on close monitoring of the clinical symptoms, laboratory tests, and serial radiologic findings. The final decision to go to surgery was made by each patient's group of surgeons. To evaluate the prognosis of ASBO patients, follow-up was conducted by the doctors who managed the patients’ treatment. The clinical parameters and lab results were collected during the medical management; authors reviewed all the enrolled patients’ records and collected data analyzed in the study retrospectively from September 2013 to April 2016.

### 2.4 Statistical analysis

Categorical variables were represented as frequency (percentage) and compared using the chi-square test or Fisher exact test as appropriate. The clinical factors associated with intestinal strangulation were analyzed by multivariate stepwise unconditional logistic regression analysis, the odds ratios (ORs), and 95% confidence intervals (CIs) were represented. The log-rank test was used to compare Kaplan–Meier recurrence curves. Univariate and multivariate Cox regressions were performed to assess the hazard ratios (HRs) and 95% CIs of the possible prognostic factors, with adjustment for possible confounders. *P* < .05 was statistically significant. All the analyses were conducted with the SPSS program (version 18.0; Chicago, IL).

## 3 Results

In 398 SBO patients, a total of 110 patients were excluded. Among excluding cases, 19 cases were diagnosed as early postoperative obstruction within 1 month; 13 patients were diagnosed as malignant disease (carcinoma or GISTs); 12 cases were abdominal tuberculosis; 8 patients were mesenteric arterial embolism or thrombosis of mesenteric vein; 4 cases were abdominal cocoon. The other 54 patients were excluded due to the data were not fully obtained during the follow-up period. Finally, 288 patients were recruited for this study.

Of these 288 patients, there were 151 males (52.4%) and 137 females (47.6%) with ages ranging from 14 to 97 years old, with a median age of 54 years (45–66 years) and an average age of 54.8 ± 15.4 years. The follow-up of these 288 cases lasted from September 2013 to April 2016 without any cases of death. The longest follow-up interval lasted for 37 months, and the shortest lasted for 6 months. The total median non-recurrence time was 32.2 months. In this study, 122 patients (42.3%, 122/288) underwent a surgical procedure. Of these, 83 patiens underwent enterolysis, and the other 39 patients underwent small bowel resection and anastomosis. One hundred and Sixty-six patients (57.6%, 166/288) received conservative management. Ultimately, 37 cases were diagnosed as ASBO with a strangulating obstruction, and 251 cases were diagnosed as simple ASBO (Table 1).

**Table 1:**
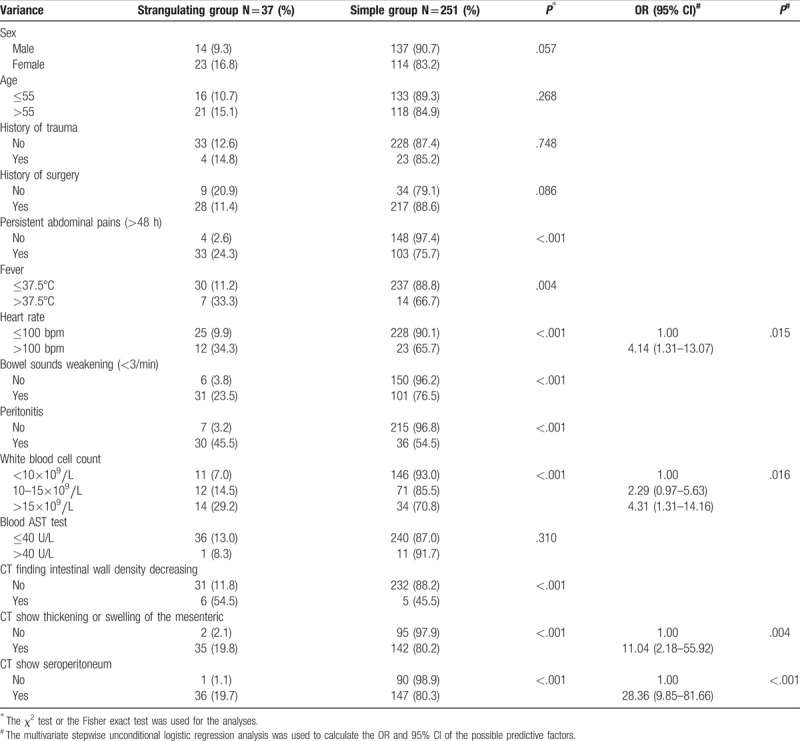
Clinical characteristics and factors associated with intestinal strangulating obstruction in ASBO patients.

### 3.1 Four independent clinical factors associated with strangulating obstruction

**Figure 1:**
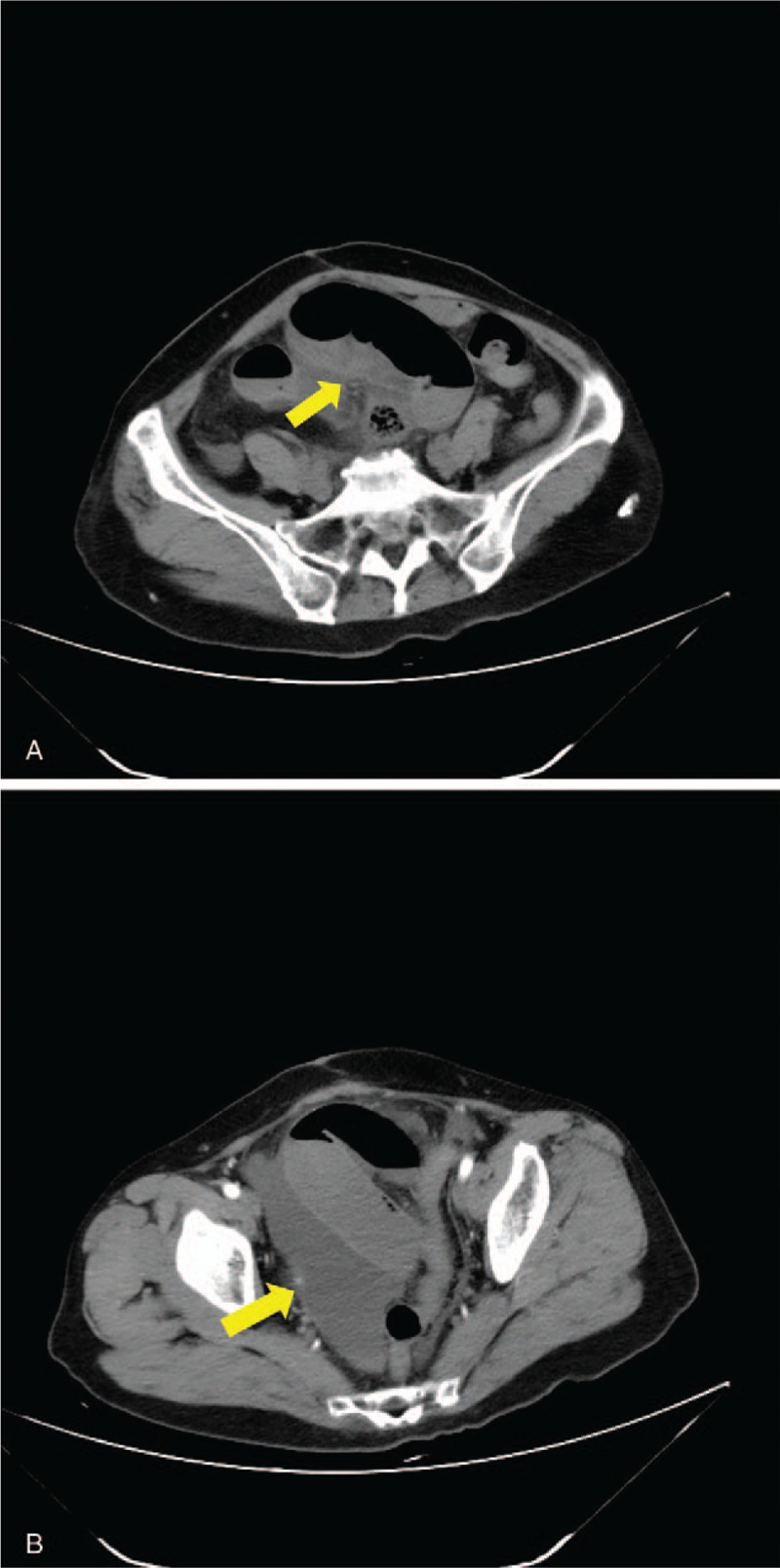
CT imaging of strangulating obstruction: (A) CT founding thickening and swelling of the mesenteric (arrow); (B) CT showing CT showing seroperitoneum (arrow).

The clinical factors associated with strangulating obstruction of the 288 cases included the following: increasing heart rate (>100 bpm), increasing WBC count (>15×10^9^/L), CT findings of thickening or swelling of the mesentery, and CT showing seroperitoneum (Fig. 1B). These clinical parameters were associated with increased risk of intestinal strangulation (*P* < .05) (Table 1).

### 3.2 Recurrence after surgery or conservative management

**Figure 2:**
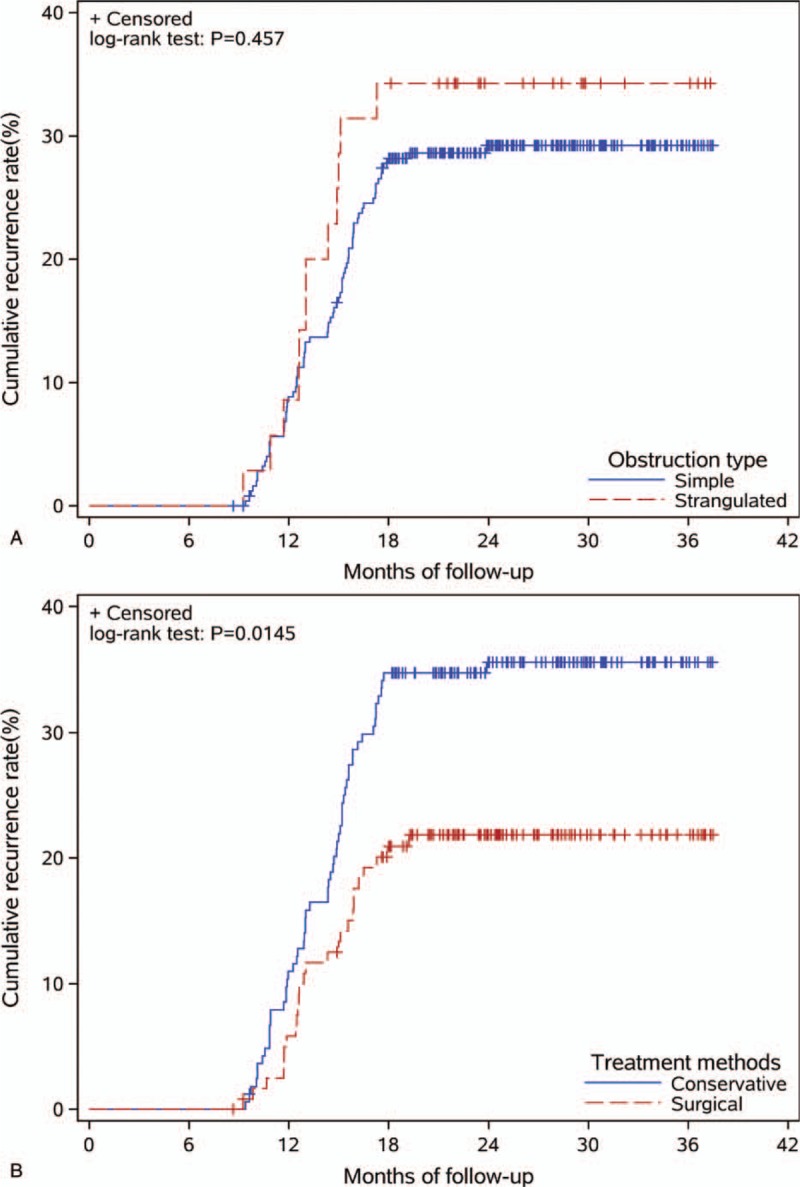
Recurrence rates comparison between different intestinal obstruction types (A) and treatments (B).

Of the 288 included ASBO patients, the obstruction recurred in 84 cases with a recurrence rate of 29.2% (84/288), and 20 of those 84 cases underwent repeat surgery [reoperation rate 23.8% (20/84)], whereas the other 64 cases underwent conservation treatment. The recurrence rate of the surgical group was 21.3% (26/122), and the median nonrecurrence interval was 19.3 months. The recurrence rate of the conservative management group was 34.9% (58/166), and the median non-recurrence interval was 33.1 months. The differences in the recurrence rates between the 2 groups were considered as statistically significant (*P* = .010) (Fig. 2A) (Table 2). The recurrent rate of the simple ASBO group was 28.6% (72/251), and the median nonrecurrence interval was 32.2 months. The recurrence rate of the strangulating obstruction group was 32.4% (12/37), and the median nonrecurrence interval was 11.5 months. The differences between these 2 groups were not considered as statistically significant (*P* = .186) (Fig. 2B). The median non-recurrence intervals of male and female patients were 31.6 months and 31.2 months, respectively. There was no statistically significant difference between the non-recurrence intervals of male and female patients (*P* = .899). The median nonrecurrence intervals between patients aged  <55 years old and ≥55 years old were not statistically significant (*P* = .673).

**Table 2:**
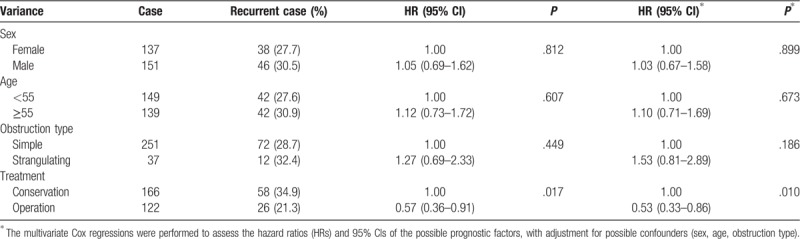
Univariate and multivariate Cox regression analysis of ASBO recurrence.

## 4 Discussion

It is said that intraperitoneal adhesions occurred in 50% to 100% of patients who undergo abdominal surgery.^[7]^ Postoperative adhesion is a significant health problem with major implications on quality of life and health care expenses. ASBO is becoming the most common type of obstruction and is accompanied by an increased abdominal reoperation rate.^[8–10]^ Adhesions may cause episodes of acute abdomen due to intestinal strangulation, therefore, predicting the need for surgical intervention among patients with ASBO is challenging. The diagnosis of intestinal strangulation is significantly important for patients to achieve better treatment results.^[11,12]^

### 4.1 Clinical factors associated with intestinal strangulation

Intestinal strangulation is associated with intestinal necrosis and severe intraperitoneal infection; therefore, timely recognition of intestinal strangulation when there is evidence of an acute abdomen is vital for surgical decision making. Our study shows that tachycardia and leukocytosis significantly associated with occurrence of intestinal strangulation in ASBO. The findings are consistent with previous investigations.^[13,14]^

Notably, present study is similar to Williams SB et al reported in 2005.^[5]^ Differently, we list 14 clinical factors for intestinal strangulation in ASBO patients, including fever, heart rate, and abdominal pain. Fever, tachycardia, and leukocytosis were classic symptoms and signs of ASBO. Thus, they were considered as associated clinical factors for intestinal strangulation rather than as risk factors contribute to abdominal adhesion. The classic symptoms and signs reflect the severity of infection in the abdomen; however, the results from typical clinical examinations usually lack detailed and accurate information specific to intestinal strangulation.

Recently, multidetector computed tomography (MDCT) has made remarkable progress in the etiological diagnosis of intestinal obstruction. Current results showed that CT findings of thickening or swelling of mesentery, and CT showing seroperitoneum significantly associated with intestinal strangulation. It has been reported that CT is a valuable imaging modality for identifying the cause of intestinal obstruction and for determining whether an intestinal hemodynamic disorder exists or not.^[15,16]^ To accurately predict intestinal strangulation in ASBO patients, surgeons should combines clinical symptoms, signs, laboratory examination and imaging findings.^[17]^

### 4.2 Risk factors for ASBO recurrence

Treatment for ASBO can be either non-operative or operative. One of the difficulties in ASBO treatment is the recurrence of intestinal obstruction after treatment. A multicenter prospective study showed that an age <40 years old, the presence of adhesions or matted adhesions, and postoperative surgical complications are the risk factors for postoperative ASBO.^[18]^ Lorentzen et al reported that the overall recurrence rate after surgically treated ASBO was 12.1%, and the risk factors for recurrence were female sex, multiple/matted adhesions, and fascial dehiscence.^[3]^

Our study indicated that ASBO patients who underwent surgical treatment have a reduced recurrence rate compared with those of non-operative group. In other words, surgical treatment decreased the recurrence of ASBO. The results are consistent with previous studies.^[6,10,19]^

Patients are managed conservatively based on the following conditions: no signs of strangulation or peritonitis, surgery > 6 weeks before onset of ASBO, and signs of resolution on admission. Various conservative methods, including nasogastric tube suction and fluid resuscitation and the administration of water-soluble contrast agents, such as gastrografin, are used. In the majority of cases, conservative treatment is an effective mean of management for ASBO.

The effective rate of non-operative management is as high as 60% to 70%, and nonoperative management can be successful in almost 90% of patients without peritonitis.^[18]^ In the present study, 166 of 288 patients who underwent conservative therapy recovered with an effective rate of 57.6%. Nevertheless, nonoperative management in parts of patients can progress to failure. Even though comprehensive conservative treatments can effectively relieve the obstruction, the abdominal adhesion does not disappear. Although these measures can effectively alleviate the symptoms of intestinal obstruction and restore function, conservative treatment does not eliminate adhesions, which can still cause recurrence in some of ASBO patients.

On the contrary, although strangulating obstructions are more likely to recur after treatment compared with simple ASBO (32.4% vs 28.6%), intestinal strangulation was not a unique independent risk factor in present study. Emergent surgery is needed for intestinal strangulation as soon as it is diagnosed and conservative management has failed. It is reported that the mortality of strangulating intestinal obstruction is 3% to 5%, and may even be as high as 10% to 30%.^[20]^ All cases of strangulation in our study underwent emergency surgery and recovered uneventfully. Surgical treatment includes both open and laparoscopic procedures, and it remains controversial whether or not laparoscopic adhesiolysis can reduce postoperative adhesion formation compared with the open abdominal surgery.^[21]^ In our center, laparoscopic surgery is suitable for selected patients who have a single band, shorter course of disease, or lower degree of intestinal distension.

Current study still have some limitations: first, the study was a retrospective single-center study, further randomized controlled trials are deeded to identify these potential factors associated with intestinal strangulation. Second, this series reporting mean follow-up were 24 months, more long-term results regarding recurrence are limited; finally, lack data of some predictive biomarkers associated with intestinal strangulating obstruction, such as levels of serum C-reactive protein and procalcitonin.

## 5 Conclusion

In conclusion, this study confirms that 2 classic clinical parameters and 2 CT finding are the clinical factors associated with intestine strangulation of ASBO, surgery could decrease the recurrence rate of ASBO.

In addition, the management of ASBO relies heavily on surgeon judgment, including decisions on how long to try conservative treatment and whether or not to use a laparoscopic or open approach if surgery is needed.^[18,22,23]^ With the development of new laboratory and imaging technology, potential factors that impact the risk of intestinal strangulation and recurrence are still need to be studied in the future.^[24–27]^

## Author contributions

**Conceptualization:** Xue-Yuan Cao, Quan Wang.

**Data curation:** Chuan Wang, Jia-Xing Song, Jing Jiang.

**Formal analysis:** Chuan Wang, Jing Jiang.

**Investigation:** Jian-Feng Mu, Shi-Dong Wang.

**Methodology:** Chuan Wang, Jing Jiang.

**Project administration:** Xue-Yuan Cao, Jian-Feng Mu.

**Resources:** Shi-Dong Wang, Jia-Xing Song.

**Software:** Chuan Wang.

**Supervision:** Xue-Yuan Cao, Quan Wang.

**Writing – original draft:** Jian-Feng Mu, Shi-Dong Wang.

**Writing – review & editing:** Xue-Yuan Cao, Jian-Feng Mu.
